# Assessing current visual tooth wear age estimation methods for *Rangifer tarandus* using a known age sample from Canada

**DOI:** 10.1371/journal.pone.0301408

**Published:** 2024-04-02

**Authors:** Grace Kohut, Robert Losey, Susan Kutz, Kamal Khidas, Tatiana Nomokonova

**Affiliations:** 1 Department of Anthropology, University of Saskatchewan, Saskatoon, Saskatchewan, Canada; 2 Department of Anthropology, University of Alberta, Edmonton, Alberta, Canada; 3 Faculty of Veterinary Medicine, University of Calgary, Calgary, Alberta, Canada; 4 Canadian Museum of Nature and Beaty Center for Species Discovery, Ottawa, Ontario, Canada; 5 Biology Department, Laurentian University, Sudbury, Ontario, Canada; Yerevan State Medical University Named after Mkhitar Heratsi, ARMENIA

## Abstract

Age estimation is crucial for investigating animal populations in the past and present. Visual examination of tooth wear and eruption is one of the most common ageing methods in zooarchaeology, wildlife management, palaeontology, and veterinary research. Such approaches are particularly advantageous because they are non-destructive, can be completed using photographs, and do not require specialized training. Several tooth wear and eruption methods have been developed for *Rangifer tarandus*, a widely distributed and long-utilized species in the North. This paper evaluates the practicality and effectiveness of three existing visual tooth wear and eruption methods for this species using a large known-age sample from several caribou populations in northern Canada (Bluenose East, Bluenose West, Dolphin-Union, Qamanirjuaq, and Beverly herds). These methods are evaluated based on: (1) the amount of error and bias between estimated and actual ages, (2) suitable and interpretable results, (3) user-friendly and unambiguous procedures, and (4) which teeth and visual features of those teeth are used to record wear and eruption status. This study finds that the three evaluated methods all have variable errors and biases, and two show extensive biases when applied to older individuals. Demographic data is simpler to generate and more flexible to report when methods allow age to be estimated as a continuous or discrete variable, rather than as age ranges. The dentition samples used by two of the previously developed methods impact their applicability to other populations of *Rangifer*. In one existing method, individuals were unavailable from some age ranges leaving gaps when assigning ages. For another *Rangifer*-ageing method, the population utilized was too distinct in morphology or diet to be used with the Canadian caribou analyzed here. Additional refinement of tooth wear and eruption ageing methods will benefit zooarchaeological research on reindeer and caribou remains.

## Introduction

Age estimation using animal dentition is an effective tool in zooarchaeology for investigating how humans interacted with fauna in the distant past [1–15]. For example, data from such estimations can be used to create age-based demographic profiles and survivorship curves that are informative about how people procured wild animals and managed domestic herds [10,12,16–19]. Ageing methods are especially valuable for key species such as caribou and reindeer (*Rangifer tarandus*). These animals are widespread across much of northern Eurasia and North America [20–23], and their remains are frequently found in abundance at archaeological sites beginning in the Late Pleistocene [e.g., 24–30]. Furthermore, reindeer and caribou, in both wild and domestic forms, continue to be essential to maintaining ways of life and well-being for many northern peoples [23,31–45].

Tooth wear age estimation is commonly used for analysing ungulate remains from archaeological sites. It is a non-destructive, inexpensive, and efficient technique that does not require specialized equipment or extensive experience, particularly when detailed methodological procedures are provided [15]. Animal teeth are subjected to wear on the occlusal surface and reduce in height over their lifetime [46–49]. Such wear progresses at a predictable rate, allowing the extent of wear to be used to estimate age at death, but always with some margin of error. For herbivores such as caribou and reindeer, the rate of wear is heavily influenced by the texture of their food, meaning that dietary differences (and any soil substrate ingested with food) lead to variation in wear patterns and rates [4,46,50–54]. Body size variation by sex and between populations also likely contributes to additional variation in these patterns. For example, the morphology of occlusal wear patterns of first and second molars have been used to differentiate between two subspecies of reindeer in Fennoscandia, mountain reindeer (*R*. *t*. *tarandus*) and forest reindeer (*R*. *t*. *fennicus*) [55]. Tooth wear methods are designed for specific species or populations due to these factors [1]. These approaches are usually paired with an examination of tooth eruption for individuals with deciduous (also known as primary) teeth in place or adult dentition still in the process of erupting [4,22].

Mandibular molars and premolars are most often used in zooarchaeological analysis because they remain in their alveoli more often than incisors and canines, and also in comparison to those in the fragile maxilla [6,10,14,56]. Methods using incisors and canines are often suitable in wildlife research and veterinary contexts because these teeth can be more easily observed in living animals [e.g., 57–59]. However, ruminant incisors and canines are smaller and morphologically simpler, and thus offer less precision for visual age estimation than post-canine teeth [1,52].

Several tooth wear ageing methods have been developed for *Rangifer tarandus*, including visual and crown height measurement approaches. Visual methods involve observing the severity of tooth wear on the occlusal surface in comparison to illustrated patterns of dentine and enamel wear or text descriptions of tooth wear characteristics. Three such methods available for this species were identified in the published literature: those by van den Berg, Loonen, and Çakırlar [60], Pasda [56], and Miller [61] which are described below. In such approaches, the analyst essentially matches observations on a tooth to the illustrations or descriptions and assigns a score representing either relative tooth wear severity or age group. Another approach for caribou and reindeer focuses on crown height measurements (often molars, but also premolars or incisors) [e.g., 52,61–65]. Measurements are considered more objective than visual assessment and the results of this approach are more easily analyzed statistically [13,66]. However, visual methods have some advantages over crown height approaches. Visual methods can be completed using photos rather than by directly accessing specimens, allowing for greater flexibility in data collection. Most importantly, these methods require no alteration to specimens other than cleaning of occlusal surfaces [60] whereas crown height cannot be measured for younger individuals without extracting teeth to reveal the full crown height [15]. Unless the alveolar bone is already fragmented, destructive procedures are necessary to extract post-canine teeth when the landmark being measured, usually the cemento-enamel junction or the bifurcation of the root, is below the alveolar bone. Chipped cusps or other damage also may prevent specimens from being properly measured, but this issue affects all tooth wear methodologies.

Visual approaches to scoring tooth wear can be effective and convenient tools for ageing reindeer and caribou remains, but their accuracy and user-friendliness commonly remain untested using other known age samples. This study evaluated published methods developed for *Rangifer tarandus* by van den Berg, Loonen, and Çakırlar [60], Pasda [56], and Miller [61] (referred to here as Methods A, B, and C, respectively) using a sample of known age caribou from Northern Canada. These methods were assessed in four ways. First, where possible, age estimations for each method were compared to known ages to assess error rates and biases. Second, we consider the format of the results from each method, including whether the estimated age was a continuous variable or an age range, and how easily the results could be interpreted. Third, the user-friendliness, clarity, and possible sources of subjectivity or misinterpretation in each method are discussed. Finally, we examine which teeth were chosen for analysis and the visual characteristics each method incorporates into its scoring models. Importantly, variations in how the three models were designed and differences in their intended uses make some numerical comparisons in the models’ performances impossible.

## Materials and methods

**Fig 1:**
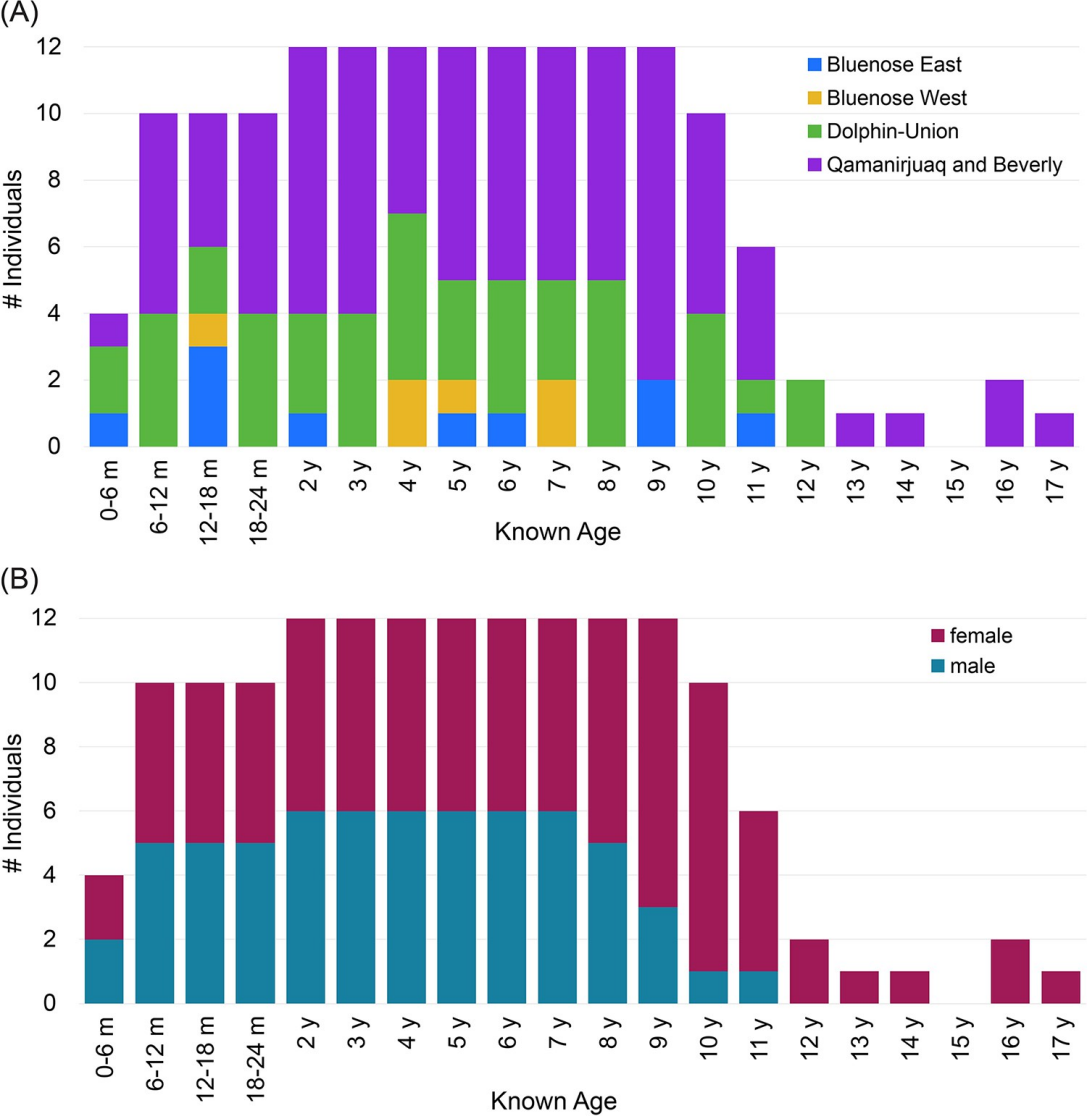
Age distribution of caribou mandible sample organized by (A) population and (B) sex.

The sample used in this study consists of left mandibles from 153 caribou, including 90 females and 63 males ranging in age between 3 months and 17 years (Fig 1A). Within this age range, only 15-year-old individuals were missing. An effort was made to maintain an even ratio of females to males for all age groups, but this was only possible for individuals up to eight years of age (Fig 1B). Male caribou and reindeer have lower life expectancies than females [22,61], and fewer older males make their way into collections. The oldest male individual available for this study was 11 years old, while females in the sample were up to 17 years of age. For both sexes, fewer caribou over age 10 were available compared to younger age classes.

Individuals in this study with fully erupted adult dentition, as well as those originally used for Methods A [60], B [56], and C [61], were aged using cementum annulation. These cementum ages are referred to here as known ages. When not feasible to track individual animals from birth to death and collect their remains (e.g., virtually all wild ungulates), cementum annulation is frequently employed in age estimation. This method introduces some error but is nonetheless considered the most accurate skeletal ageing method currently available [4,51,67–69]. For example, using cementum annulation Veiberg and colleagues [69] found ages of semi-domestic reindeer (*R*. *t*. *tarandus*) in Norway were assigned correctly in 54% of cases, with 89% having age estimates within one year of true age. In another study, Svalbard reindeer (*R*. *t*. *platyrhynchus*) ages were correct in 71% of cases, with 94% aged to within one year of their known ages. Ages for individuals with erupting dentition can be estimated where the calving season is brief using the known date of death and the status of tooth eruption [61].

**Fig 2:**
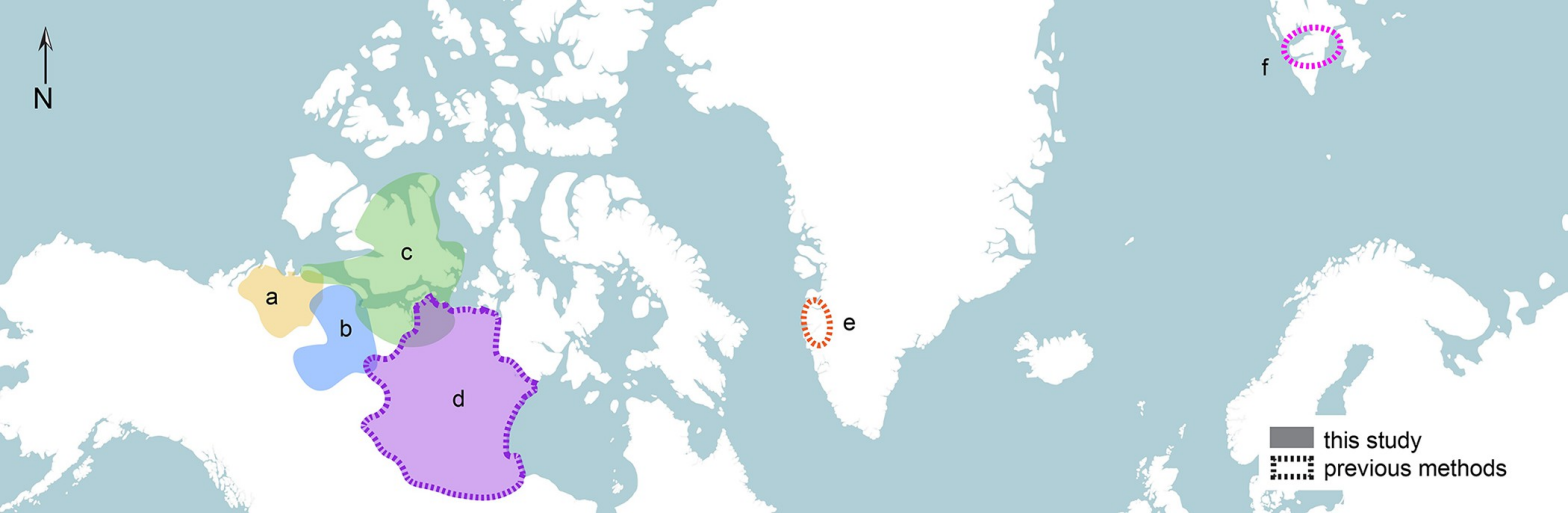
Geographic distributions of caribou and reindeer populations discussed in this study: (A) Bluenose West and (B) Bluenose East caribou herds [71,72], (C) Dolphin-Union caribou [73], (D) Qamanirjuaq and Beverly caribou herds [61,70,74], (E) Sisimiut caribou [56,75], and (F) Svalbard (Nordenskiöld Land) reindeer [60]. Basemap made with Natural Earth.

*Rangifer tarandus* in this study belong to several populations in Northern Canada (Fig 2). Ninety-two (57 females, 35 males) barren-ground caribou (*R*. *t*. *groenlandicus*) were from the Qamanirjuaq and Beverly Herds. Whether these individuals belong to the Qamanirjuaq or Beverly herd is unknown. Since these herds share similar ranges which overlap during the winter months and are of the same subspecies [61,70], it was considered unnecessary to differentiate between the two. Their mandibles were collected by the Canadian Wildlife Services (CWS) between 1966 and 1968 and are curated at the Canadian Museum of Nature, Gatineau, QC, Canada. Adult ages were determined through cementum annulation by F. Miller and CWS personnel, and all or some were used in the original development of Method C [61]. The study sample also includes 45 (29 female, 16 male) caribou from the Dolphin-Union Herd (*R*. *t*. *groenlandicus x pearyi*), 10 (4 female, 6 male) from the Bluenose East Herd (*R*. *t*. *groenlandicus*), and 6 (all male) from the Bluenose West Herd (*R*. *t*. *groenlandicus*). Mandibles from these three herds were collected through hunter sampling programs by the Kutz Research Group, Faculty of Veterinary Medicine, University of Calgary, between 2008 and 2019. They are curated at the Zooarchaeology Lab, Department of Anthropology, University of Saskatchewan in Canada. For this latter group of specimens, adult ages were assessed through cementum annulation of incisors by Matson’s Laboratory (Manhattan, MT, USA). All mandibles in this sample are listed in the S1 Table.

A total of 912 mandibular molars and premolars were present in the 153 mandible sample (Table 1, S1 Table). This included 92 deciduous premolars, 383 permanent premolars, and 437 molars. Unerupted teeth (25 P_2_, 23 P_3_, 25 P_4_, 0 M_1_, 3 M_2_, and 16 M_3_; 98 total) could not be scored for wear and were recorded as absent (not included in Table 1 counts). Seven additional teeth were missing due to irregular loss. One dP_2_ (CMN-39169), P_2_, (CMN-39516), and two M_3_ (CMN-39112 and DU-210) were lost at or around the time of death (alveoli are open without remodeling). One dP_2_ (CMN-38606) was severely fractured with most of the cusp missing. One P_3_ (BW-08-91) was lost antemortem (alveolus has remodelled) and the M_3_ was missing from one mandible (CMN-39056) with bone deformation and resorption surrounding the M_3_ alveolus.

**Table 1:** Number of teeth available for analysis in caribou mandible sample.

Population	dP_2_	dP_3_	dP_4_	P_2_	P_3_	P_4_	M_1_	M_2_	M_3_	Total
Qamanirjuaq and Beverly	13	14	15	80	82	81	92	92	81	551
Dolphin-Union	11	11	10	36	37	37	45	43	38	268
Bluenose East	5	5	5	5	6	5	10	9	9	59
Bluenose West	1	1	1	5	4	5	6	6	6	35
Total	30	31	31	126	129	128	153	150	134	912

Tooth wear was observed in photos taken with a mirrorless camera from occlusal, lingual, and buccal views. All assessments were completed by the same person (G. Kohut). Teeth were evaluated blindly (without known age data) to reduce observer bias. Data were entered and processed with Microsoft Excel. Unless otherwise noted, ages of caribou and reindeer were considered in “birthday years” and rounded down to the nearest integer (i.e., a 3.8-year-old is a three-year-old, not a four-year-old).

### Method A

Van den Berg et al.’s [60] method (Table 2) was developed for Svalbard reindeer (Fig 2) and is similar in design to Grant’s [5] widely used method for cattle, sheep/goats, and pigs. Svalbard reindeer (*R*. *t*. *platyrhynchus*) dentition from 151 known age individuals (292 mandibles) ranging from 0–15 years of age were used to create a series of black and white illustrations of the occlusal view of dentine and enamel for dP_4_, P_4_, M_1_, M_2_, and M_3_. Two versions are provided: the Svalbard or Absolute scheme and the uncalibrated or Relative scheme for other reindeer populations. The Svalbard version provides an age estimate in years and is meant for use solely with Svalbard reindeer. This population is genetically isolated and has adapted to the high-arctic Svalbard Archipelago with short limbs, small bodies, and small head sizes, differentiating them from most other subspecies in both morphology and diet [76,77]. The uncalibrated or Relative scheme is recommended by the authors for non-Svalbard populations, and this version provides an arbitrary score that ranks individuals relatively but does not provide an age estimate [60].

**Table 2:** Tooth wear age estimation methods for caribou and reindeer evaluated in this study.

Method	Source	Population	Subspecies	Region	Sample Size (# of Individuals)
Method A	van den Berg, Loonen, and Çakırlar [60]	Svalbard Reindeer	*R*. *t*. *platyrhynchus*	Svalbard Archipelago	151
Method B	Pasda [56]	Sisimiut Caribou	*R*. *t*. *groenlandicus*	Greenland	63
Method C	Miller [61]	Qamanirjuaq and Beverly Herds	*R*. *t*. *groenlandicus*	Northern Canada	999

To test these methodological schemes, age estimation for our study sample first involved the application of van den Berg, Loonen, and Çakırlar’s [60] uncalibrated or Relative teeth wear progression scheme. Each dP_4_ or P_4_ (whichever is present) and M_1_, M_2_, and M_3_ were assigned a Tooth Wear Stage (TWS) letter based on the closest match between the occlusal pattern of dentine and enamel and the black and white illustrations. If a tooth was in the process of erupting, it was assigned a TWS code of “C, perforation in Crypt visible; V, tooth Visible in crypt; E, tooth eruption through bone; H, tooth almost halfway between bone and full height; [and] T, tooth (almost) at full height but unworn” [60]. Absent teeth or those that could not be assessed (e.g., broken crowns) were omitted. Teeth not visible because they had not yet erupted were scored as zero.

Because the Relative scheme does not produce age estimations, additional steps were taken. Specifically, the TWS scores were calibrated using the known age samples compiled for this study. Our approach in this step followed the calibration process utilized with the Svalbard Absolute scheme [60]. Namely, an age assessment was made for each tooth TWS by calculating the mean known age of all mandibles in the current study sample fit that a particular TWS. These calibrated scores were rounded to the nearest 0.5 to remain consistent with the original calibrated method and are referred to as the “calibrated version” of Method A. The mandible wear stage (MWS) was calculated as the mean TWS score for all teeth present in each mandible. Unerupted teeth were counted towards the total number of teeth while missing or damaged teeth were not. The estimated age in years equals the MWS. This process of testing Method A is not ideal, as the model was calibrated on the same specimens that were then aged, and the results compared to the known ages. This somewhat circular procedure should reduce errors in age assessment compared to the other models tested. At the same time, this step makes it problematic to directly compare these error rates with those of the other methods. Regardless, this procedure allowed us to assess the usability of the relative scheme in a similar way among all three methods.

The second assessment of Method A involved ageing our sample using the Absolute scheme as if the sample were composed of Svalbard reindeer. As mentioned, the use of the Absolute scheme with non-Svalbard reindeer was not recommended by the study authors for several valid reasons [60]. Our utilization of the Absolute scheme on non-Svalbard reindeer helps demonstrate this point, namely that it is problematic to apply an ageing method designed for a highly distinct population more broadly. Further, this step also allowed for an assessment of the practicality of employing the model realistically, albeit not on its intended target population.

### Method B

Pasda’s [56] ageing method (Table 2) was developed on Sisimiut caribou (*R*. *t*. *groenlandicus*) from Greenland (Fig 2). The dentition of 63 reindeer of known age ranging from 0–14 years (0–177 months) was used to establish criteria for estimating age using mandibular molar and premolar wear patterns. These reindeer died of natural causes and their remains were collected after death. Cementum annulation examination was carried out by the study author [56,75]. Teeth were graded visually based on the severity of wear or tooth eruption and replacement. A series of representative photos of dentition from the occlusal and buccal views were also provided. Pasda cautions that “this classification was subjective and qualifies as a rough estimate” [56, p. 33]. While Sisimiut caribou should be relatively comparable to the populations used in this study given that they belong to the same subspecies, Pasda [56] notes that reindeer in this region (Kangerlussuaq) are known for having accelerated tooth wear relative to more southern populations in Greenland, attributed to a courser diet.

Method B involved visually scoring the extent of wear of each tooth using the scoring criteria provided in Table 3 and then assigning age ranges using the descriptions available in the published text [56, tbl. 8]. Ages in the current study were estimated following the published instructions, but some additional procedures also were implemented. First, following the original protocol, the premolars and molars in each mandible were assigned grades: no wear, slight wear, moderate wear, heavy wear, and very heavy wear following Table 3 [56]. Additionally, we recorded the status of eruption, if applicable, and whether premolars were deciduous or permanent.

**Table 3:** Description of tooth wear grades used in Method B, as outlined in Pasda [56, p. 33].

Wear Grade	Description
none	no wear
slight	“polished surface and the dentine was exposed at the cusps (e.g., individual no. 18 or 34)”
moderate	“dentine of teeth … showed greater and more even exposure (e.g., individual no. 8 or 170).”
heavy	“teeth already irregularly worn (e.g., individual no. 11). Frequently the M1 was abraded down to the roots while the M_2_ and M_3_ showed relatively even exposure (e.g., individual no. 13).”
very heavy	“only the root remained intact with some fragments of teeth (e.g., individual no. 173).”

Second, using the teeth grades, individual mandibles were assigned to age ranges following the study protocol [56, tbl. 8]. However, multiple age ranges were usually applicable to each mandible. A procedure for assigning these age categories was implemented: when two or more age ranges applied to one individual, the overlapping range was used as the final estimated age range. For example, if the M_1_ and M_2_ are both moderately worn, their age ranges in the original study protocol, 31–71 and 36–71 months respectively, were combined into an estimated age range of 36–71 months. For instances where two or more criteria applied to one mandible but have age ranges that do not overlap (for example, M_3_ erupting at 13–18 months and M_1_ moderately worn at 31–71 months), one of the ranges was disregarded. These choices were made without consulting the known age data. In cases where two or more age ranges overlap but one did not, the non-overlapping age range was omitted. If one of the non-overlapping criteria involved tooth eruption, that range was chosen since tooth eruption is more reliable for ageing than wear. Finally, if the above rules could not be used to choose between non-overlapping ranges, the criterion with a more clear and easily distinguished description of tooth wear was used, though these choices were admittedly subjective.

### Method C

Miller’s [61] caribou ageing study (Table 2) involved 999 known-age individuals between 0–17 years belonging to the Qamanirjuaq (published as Kaminuriak) and Beverly caribou herds (*R*. *t*. *groenlandicus*). These herds migrate in overlapping ranges in northern Canada (Fig 2). A small portion of Miller’s caribou mandibles (n = 92) collected between 1966–1968 was also used in the present study. In Miller’s study, mandibular dentition was used to generate text descriptions of tooth wear and eruption for complete rows of teeth including molars, premolars, canines, and incisors, though canines and incisors are referenced less often [61, p. 15–18]. Incisors and canines were not used in the present study as these teeth were not available for most specimens. Descriptions are available for 19 age categories, provided in months, ranging from birth to 10 years, including a 10+ years age category. Eleven of those age categories are within the first three years and are based on tooth eruption. Tooth eruption data is provided in a table [61] which reports the percent frequency of occurrence of whether each tooth type is unerupted, deciduous, partially, or fully erupted for ages between zero and 29 months.

Specific instructions for applying Miller’s data to estimate age were not provided, requiring that specific steps be outlined here. First, to establish an approximate age range, each mandible was compared to either the tooth eruption table [61] or reference photos, depending on whether tooth eruption was ongoing at death. Second, using the approximate age range as a starting point, each individual was assigned a more specific age estimate based on the tooth wear and eruption descriptions. For cases where one mandible fit the description for more than one age (which occurred frequently), the closest fitting description was chosen. Specifically, each individual was placed into age categories utilized by Miller [61], which were 1, 2, 3, 4–6, 6–9, and 10+ years. Miller [61] cautions that estimated ages are inaccurate from approximately four years and older, and these broader age categories are more appropriate. In our evaluation of method C, we excluded the 92 mandibles examined here used by Miller in the initial development of his method. Method C was thus applied only to the 61 individuals belonging to the Dolphin-Union, Bluenose East, and Bluenose West populations.

## Results

To present and interpret the study outcomes consistently, two known age category schemes were employed. First, the initial two years of life were divided into six-month intervals, followed by one-year intervals for ages two to 17. Second, individuals were divided into three age groups (0–2, 3–9, and 10+ years) to investigate trends according to general young, adult, and old adult life stage categories for reindeer and caribou [17,61]. The division between young and adult at three years was based on sexual maturity and adult tooth eruption completion, the latter occurring at ~29 months [61,78–81]. The beginning age for old adults is approximately when *Rangifer* become reproductively unviable (particularly when females stop producing calves) and body mass begins to decrease, though this varies by population [65,78,80,82–84]. Miller [61] also employs a broad 10+ year age category in his analyses, which we adopt here so that this age category can be reported consistently between Methods A, B, and C.

Assessing error is essential to understanding how accurately methods estimate age. Because the estimated age for Method A was a continuous variable, Method B provided a range, and Method C involved discrete or range values, direct statistical comparison between methods was impossible. Where estimated ages were a discrete value (Method A and Method C for 0–9 year olds), error was calculated as the difference between estimated age and known age for each individual. For methods that produce age ranges (Methods B and C), error was calculated as the difference between the known age and either the upper or lower limit of the estimated age. As age ranges were more likely to result in low or no error estimations (the known age falls within the estimated age range), greater accuracy was expected compared to methods where age is estimated as a continuous variable. The mean error (*ME*) was calculated for all three methods, here only to indicate whether ages were underestimated (negative value) or overestimated (positive value). The mean absolute error (*MAE*) was used to quantify the difference between estimated and known age. This approach prevented individual positive and negative errors from cancelling each other out. Given the differences in variable type for the three methods described above, the mean errors are not directly comparable.

Ages were estimated and the *ME* and *MAE* calculated for the mandibles in this sample following Methods A, B, and C. Estimated ages for each individual using Method A (calibrated, uncalibrated, and Svalbard versions), Method B, and Method C (discrete ages and age categories) are provided in S1 Table.

### Method A

A calibrated version of Method A was successfully created based on the 153 caribou mandible sample in this study, with TWS values provided in Table 4. Most TWSs could be assigned, but there were some gaps, particularly at younger ages such as dP_4_ TWS a and b, P_4_ TWS E, and older ages such as P4 TWS j and over, M_1_ TWSs m and o, M_2_ TWSs m, and M_3_ TWS l and k. These gaps exist because no teeth matched the illustrated wear patterns. It is also notable that lengths of time between TWSs were not equal and some ages were less represented (e.g., 4, 6, and 8 years) in the calibrated TWS scheme. The P_4_ TWS f score seemed out of order with a value of 16 falling between 10 and 12.

**Table 4:** Method A calibrated tooth wear stage (TWS) values based on van den Berg, Loonen, and Çakırlar [60].

TWS	dP_4_	P_4_	M_1_	M_2_	M_3_
C				0.5	1
V		2		0.5	1.5
E				1	1.5
H		2		1	2
T		2	0.5	1	2
a		3	1	1.5	2.5
b		5.5	1.5	2	3.5
d	0.5	7.5	3	4	4.5
e	1	10	5.5	5	7
f	1	16	7	7.5	9
g	1.5	12	7.5	9.5	10.5
h	1.5	15	9.5	10.5	14.5
j			9.5	13.5	
k			11	16	
l			10	16.5	16.5
m					
n			16.5	17	
o					
p			17		

**Fig 3:**
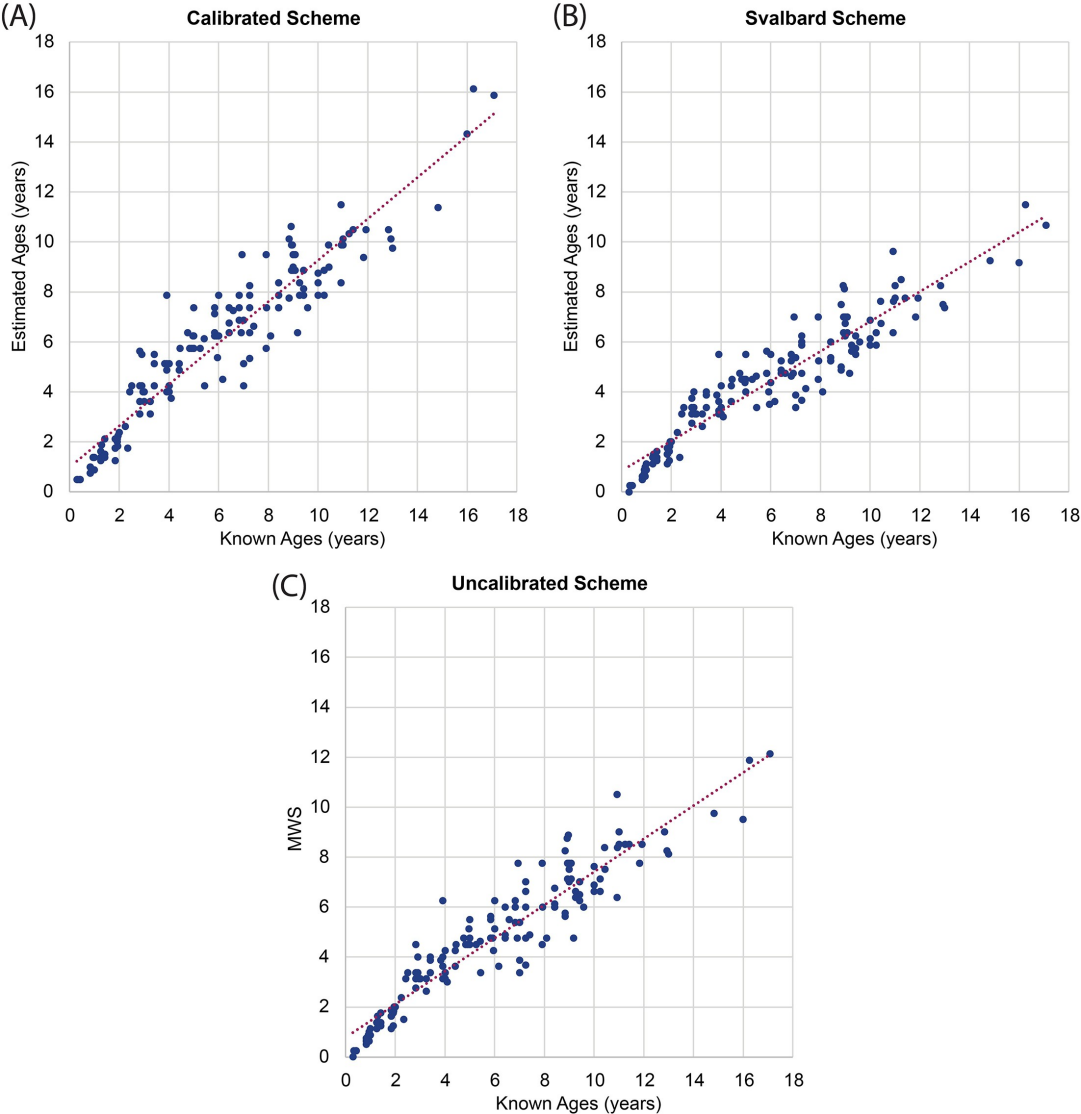
Method A age estimation scatter plots using the (A) calibrated version, (B) Svalbard version, and (C) uncalibrated version (MWS values are arbitrary values without units).

As expected, the calibrated and Svalbard versions of Method A provided contrasting age estimation results, especially for older individuals. The calibrated version results ranged from 0.5 to 16.1 years while the Svalbard version ranged only between 0.0 and 11.5 years. Also, as anticipated, the calibrated version performed well for most ages. For adults between 3–9 years (known-age) the *MAE* is 0.99 years and for ages 10+ the *MAE* equalled 1.63 years (Table 5). Based on *ME* and the scatter plot (Fig 3A), ages 2–6 tended to be overestimated while ages seven and over were more frequently underestimated.

**Table 5:** Mean error and mean absolute error for Method A Svalbard and calibrated versions (applied to 153 mandible samples).

	Method A Svalbard		Method A Calibrated	
Known Age	*ME* (years)	*MAE* (years)	*ME* (years)	*MAE* (years)
0–2 years	-0.03	0.3	0.38	0.47
3–9 years	-1.57	1.66	0.2	0.99
10+ years	-4.16	4.16	-1.58	1.63
0–5 months	-0.18	0.18	0.13	0.13
6–11 months	-0.22	0.23	0.05	0.13
12–17 months	0.05	0.14	0.22	0.25
18–23 months	-0.31	0.32	0.02	0.21
2 years	0.35	0.52	1.15	1.24
3 years	0.03	0.45	1.17	1.19
4 years	-0.07	0.67	1.16	1.16
5 years	-1.04	1.12	0.66	0.96
6 years	-1.51	1.52	0.5	0.88
7 years	-2.37	2.37	-0.64	1.2
8 years	-2.58	2.58	-0.15	0.96
9 years	-3.13	3.13	-0.84	0.99
10 years	-3.5	3.5	-1.38	1.49
11 years	-3.57	3.57	-1.29	1.29
12 years	-5	5	-2.57	2.57
13 years	-5.63	5.63	-3.25	3.25
14 years	-5.58	5.58	-3.46	3.46
16 years	-5.79	5.79	-0.9	0.9
17 years	-6.42	6.42	-1.21	1.21
Total	-1.47	1.64	0.02	0.96

The Svalbard version did not effectively model tooth wear for the barren-ground and Dolphin-Union caribou in this sample for most adult ages, as anticipated by the model’s designers [60]. The scatter diagram in Fig 3B shows a general trend of underestimating age after approximately four years old, with the oldest estimated age coming to 11.5 years (known to be 16 years old). The *MAE* remained low, less than one year, between 0–4 years old (Table 5). After a known age of four years, the discrepancy between estimated and known age increased with a *MAE* of 1.5 years (*ME* = 1.5 years) at six years old and *MAE* of 2.6 years (*ME* = -2.6 years) at eight. For caribou 10 years and older, the *MAE* was 4.16 years (*ME* = -4.16 years). Interestingly, the Svalbard version estimated the ages of younger individuals more accurately and with less bias than the calibrated version. For ages 0–2 years, the *ME* from the Svalbard version is only -0.03 compared to the calibrated *ME* of 0.38 years and the *MAE* from the Svalbard version was slightly lower (0.30 years) compared to the calibrated one (0.47 years). Based on visual inspection of the scatterplots (Fig 3), the calibrated scheme for Method A yielded a similar degree of precision to the Svalbard and uncalibrated versions. The scatter plot data points were similarly diffuse for all three versions.

### Method B

Method B estimated age categories for the sample of 153 mandibles were between three months and seven years (Table 6). These ranges cover various lengths of time, some of which overlap with each other, and two were discrete values (0.5 years/six months and seven years/84 months). Sixty-nine (45.1%) mandibles were assigned into correct age categories while 35 (22.9%) were one year off, 14 (9.2%) were two years off, and 35 (22.9%) were off by three or more years. For young caribou 0–2 years old (known age) the *MAE* = 0.19 and the *ME* = -0.06 indicated low bias and error. The error was higher for adult animals three years and over, with a high degree of bias (*ME* = -5.73, *MAE* = 5.73) (Table 7). For older individuals, age was more noticeably underestimated (for 7–17 years known age: *ME* = -3.72, *MAE* = 3.72). This was largely because individuals with known ages of 8–17 years were placed in the 3.5–6 year, 5.5–6 year, and 7 year categories. Estimated age ranges were much closer to expected values for individuals seven years and younger (*ME* = -0.29, *MAE* = 0.35) compared to ages eight years and over (*ME* = -4.30, *MAE* = 4.30).

**Table 6:** Method B estimation ages (applied to 153 mandible samples). Highlighted cells indicate overlapping known and estimated age ranges.

	Estimated Age Ranges										
Known Ages	3–5 m	6 m	7–13 m	12–18 m	12–35 m	13–18 m	3–3.5 y	3.5–6 y	5.5–6 y	7 y	Total
0–5 m	2	2									4
6–11 m		1	9								10
12–17 m				2		8					10
18–23 m					1	9					10
2 y					1	3	6	2			12
3 y					2		4	6			12
4 y							2	10			12
5 y							1	11			12
6 y								12			12
7 y								12			12
8 y								10		2	12
9 y								12			12
10 y								8	1	1	10
11 y								6			6
12 y										2	2
13 y								1			1
14 y										1	1
16 y										2	2
17 y										1	1
Total	2	3	9	2	4	20	13	90	1	9	153

**Table 7:** Mean error and mean absolute error for Method B (applied to 153 mandible sample).

Known Age	*ME* (years)	*MAE* (years)
0–2 years	-0.06	0.19
3–9 years	-1.18	1.18
10+ years	-5.73	5.73
0–5 months	0.04	0.04
6–11 months	-0.03	0.03
12–17 months	0.01	0.01
18–23 months	-0.34	0.34
2 years	0.05	0.39
3 years	-0.04	0.1
4 years	-0.08	0.08
5 years	-0.16	0.16
6 years	-0.61	0.61
7 years	-1.45	1.45
8 years	-2.57	2.57
9 years	-3.31	3.31
10 years	-4.39	4.39
11 years	-5.49	5.49
12 years	-5.88	5.88
13 years	-7.08	7.08
14 years	-7.83	7.83
16 years	-9.13	9.13
17 years	-10.08	10.08
Total	-1.53	1.57

The results of Method B, especially for older age categories, were affected by conflicting tooth wear criteria. For example, CMN-39516 was estimated to be 84 months (7 years) because it meets the criteria “premolars and M_3_ moderately worn” (43–84 months) and “crowns of the molars and premolars show heavy, sometimes irregular wear” (>84 months) [56]. However, “M_1_ shows excessive wear, only roots remaining in some cases” (>91 months) also applied to this specimen. The known age of this individual is 17 years. Because the two overlapping ranges were combined and kept (resulting in an estimated age range of 43–84 months) the higher range (>91 months) was omitted, contributing a high degree of error. Five other sets of conflicting ageing criteria were also found to be present in our sample, affecting the ageing of 32 specimens (Table 8). Combined, these individuals show an *ME* = -1.8 years and *MAE* = 1.8 years.

**Table 8:** Method B conflicting tooth wear criteria [56, tbl. 8] and associated ME and MAE.

# of Conflicts	Conflicting Criteria	Omitted	*ME* (years)	*MAE* (years)
12	“premolars and M3 show slight wear “[36–42], “premolars and M3 moderately worn” [43–84]	[36–42]	0	0.2
10*	“M2 slight wear” [12–35], “premolars and M3 show slight wear “[36–42]	[36–42]	-0.2	0.2
7	“M1 heavily worn” [>79], “M2 moderately worn” [36–71]	[>79]	-4.2	4.2
2*	“M3 erupting; deciduous premolars moderately or heavily worn…” [13–18], “M1 moderately worn” [31–71]	[31–71]	-0.5	0.5
2	“premolars and M3 moderately worn” [43–84], 20 [>108], (one also meets 11 [31–71] and “M2 moderately worn” [36–71])	[>108]	-7.3	7.3
1	“premolars and M3 moderately worn”[43–84], “M1 shows excessive wear, only roots remaining in some cases” [>91], “crowns of the molars and premolars show heavy, sometimes irregular wear” [>84]	[>91]	-10.1	10.1

* two mandibles exhibited two sets of conflicting tooth wear criteria and are counted twice in this table

### Method C

**Fig 4:**
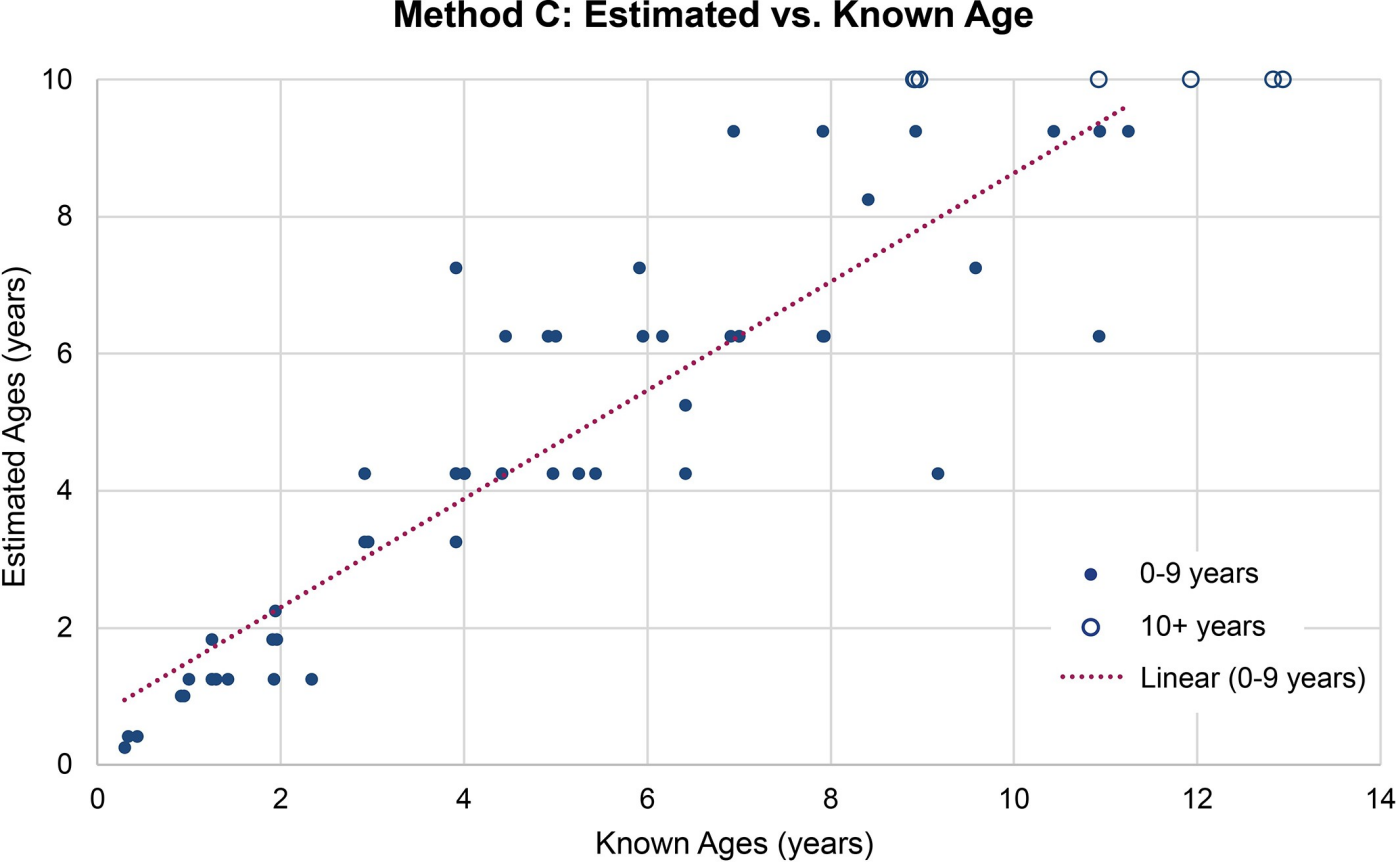
Method C age estimation scatter plot. Estimated age values in months provided in Miller [61] are used rather than years (e.g., 39 months/3.25 years instead of 3 years). The linear line of best fit applies only to individuals nine years and younger since older animals fall into a 10+ year category and cannot be modelled.

The estimated ages for the 61 mandibles evaluated by Method C ranged from three months to 10+ years. The estimated age values (before being placed into age categories) are visualized as a scatter plot in Fig 4. Known and estimated age for individuals between 0–9 years followed a positively correlated linear relationship, with the data points becoming more diffuse after roughly four years of age. The results from Method C following the age categories recommended by Miller [61] are summarized in Table 9. This comparison showed that 34 (55.7%) mandibles were categorized correctly while 18 (23.5%) were one year off, seven (11.5%) were two years off, and two (3.3%) were incorrect by three or more years. This pattern parallels that in the scatter plot generated for the estimated age values (Fig 4). The overall *ME* of -0.17 suggested a small overall underestimation bias, though the *ME* is nearly always positive until five years (known age) and negative at six years and older (Table 10).

**Table 9:** Method C estimation ages (applied to 61 caribou mandibles from Dolphin-Union, Bluenose East, and Bluenose West populations only). Highlighted cells indicate overlapping known and estimated age ranges.

	Method C Estimated Age Categories (years)							
Known Ages	0	1	2	3	4–5	6–9	10+	Total
0–5 months	3							3
6–11 months		4						4
12–17 months		6						6
18–23 months		3	1					4
2 years		1		2	1			4
3 years				1	2	1		4
4 years					4	2		6
5 years					2	3		5
6 years					2	3		5
7 years						5		5
8 years						2	3	5
9 years					1	1		2
10 years						3	1	4
11 years						1	1	2
12 years							2	2
Total	3	14	1	3	12	21	7	61

**Table 10:** Mean error and mean absolute error for Method C (applied to 61 caribou mandibles from Dolphin-Union, Bluenose East, and Bluenose West populations only).

Known Age	*ME* (years)	*MAE* (years)
0–3 years	0.06	0.27
3–10 years	-0.07	1.18
10+ years	-1.19	1.19
0–6 months	0.01	0.05
6–12 months	0.07	0.07
12–18 months	0.10	0.17
18–24 months	-0.14	0.30
2 years	0.22	0.76
3 years	0.84	1.17
4 years	0.46	0.75
5 years	0.14	1.01
6 years	-0.32	1.28
7 years	-0.70	1.24
8 years	0.67	0.74
9 years	-3.63	3.63
10 years	-1.89	1.89
11 years	-1.00	1.00
12 years	0.00	0.00
Total	-0.17	0.87

## Discussion

As discussed, since the estimated age variables produced by each of these methods are different types, it is not possible to directly compare the results quantitatively. In general, all methods produced relatively small *ME* and *MAE* values for young *Rangifer tarandus* (known age = 0–2 years) compared to those for adults and old adults. Tooth eruption played the primary role in all three methods in ageing individuals of this age range. The timing of tooth eruption can be more precisely defined than the extent of tooth wear, and, at this early life stage, variation in the rate of wear is relatively minimal [46,60,61]. However, error approaching half a year, as seen in the calibrated version of Method A (*ME* = 0.38, *MAE* = 0.47) for 0–2 year-olds (Table 5), would have limited interpretive value for inferring the seasonality of death, a common but often potentially problematic zooarchaeological practice with juvenile ungulates [7,10,85].

All three methods presented some challenges when estimating adult ages. The results from applying the Svalbard version of Method A to the caribou in this sample fully support van den Berg et al.’s [60] argument that the Svalbard Absolute ageing scheme should not be used with other *Rangifer* populations. As mentioned, Svalbard reindeer are smaller, have a different morphology than barren-ground caribou, and live in a more severe, high-arctic habitat. The tendency of this method to underestimate ages of adults suggests that Svalbard reindeer teeth wear down much faster than in the known age sample compiled for this study. In contrast, while the calibrated version would almost certainly produce more accurate results for *Rangifer* populations similar to those included here, it has only been assessed using the same sample it was calibrated with, and not all illustrations have been assigned TWS values. This version of Method A should not be considered tested for accuracy and bias.

Method B began to underestimate adult age starting around seven years. Individuals three to six years old were estimated with relatively low error (*ME* = -0.23; *MAE* = 0.24), but increased considerably for 7–9 year olds (*ME* = -2.44; *MAE* = 2.44). Specifically, all but two 7–9 year old individuals (n = 34/36) were placed in the 3.5–6 year category (Table 6). This pattern occurred primarily because no individuals were estimated to be older than seven years (84 months). It is uncertain if differences in the rate of tooth wear or dental morphology between the Canadian caribou in this sample and Sisimiut caribou from Greenland contributed to estimation error. Method B included several open-ended older individual age categories: >79 months, >67 months, >91 months, > 84–96 months, and > 173 months [56]. However, individuals matching these open-ended older age categories also matched other criteria that overlapped in age range. This bounds the upper limit of the estimated age for such individuals, often resulting in underestimation of their ages in our comparisons. The oldest age category in Method C was also an open-ended age range. It combined all older animals into a broad 10+ year category if “all molariform teeth were very worn: they were on a more or less even plane, and the buccal-lingual plane was nearly horizontal” and “the infundibulum between the anterior cusps of the M_1_ was nearly obliterated or absent in some specimens” [61, p. 18]. This approach results in fewer errors but also far less discrete age estimations for older individuals.

The ability to interpret age estimation results and assign meaningful age categories is crucial. Van den Berg and colleagues [60] did not propose age categories to use with their method. However, since the estimated age produced by Method A for the Svalbard or calibrated versions was a continuous variable, the results can be divided into any age category pattern desired. The uncalibrated version, which was designed for the relative ageing of assemblages, cannot be used in this manner because it is unknown what MWS values divide a sample into meaningful life stages. Relative age comparisons have interpretive value, but there are many cases where age estimates as continuous values, even for use within age categories, would also be useful.

The Method B age categories are very challenging to transform into a demographic profile because they overlap with each other, some representing very similar ranges, such as 12–18 months, 13–18 months, and 12–35 months. They also cover different range sizes; for example, nine individuals fell into the discrete 84 month (seven year) old category while 90 were assigned the 43–71 month (3.5–6 year) category. These results would require some reorganising before fitting into a histogram or other plot. The results from Method B are less flexible and more difficult to interpret than the other two methods because of these complicating factors.

Further, the age categories for Method B reflect the relatively limited Sisimiut caribou sample available. For example, no individuals with ages between 18–31 months were available [56], so criteria that involve later stages of tooth eruption (M_3_ and premolars erupting, M_1_ with slight wear, incomplete root development, and any molars or premolars showing no wear) end in the scheme at 18 months. In the Qamanirjuaq and Beverly caribou of the same subspecies (*R*.*t*. *groenlandicus*), the M_3_ and permanent premolars continue to erupt until ~27 months [61]. Assuming that Sisimiut caribou tooth eruption extends sometime later than 18 months, estimated ages based on this method may be underestimated for individuals with erupting M_3_ and premolars. Our results indicated that caribou with known ages of 18–29 months were underestimated with a *ME* of -0.35 years, which was notably high relative to the low degree of bias seen in 12–17 month olds (*ME* = 0.01) and three year olds (*ME* = -0.04) using this method.

Miller’s age categories for Method C could be used to reconstruct demographic profiles in wildlife management or zooarchaeological contexts [61]. By using increasingly broader age categories, Method C acknowledges that the accuracy of tooth wear ageing diminishes at older ages. However, this method would not adjust well if other age categories are desired, especially for individuals over 10 years old.

Assessing the clarity and user-friendliness of the instructions for each method is far more subjective than the assessments above but nonetheless warrants discussion. Assigning TWS scores according to Method A was intuitive and efficient to complete, in large part because the method relies on visual recognition of occlusal tooth wear patterns rather than reference to text descriptions. However, some teeth analyzed looked like they belonged between two TWSs or showed characteristics of more than one illustration, making selection of some TWS values ambiguous. Method B was also relatively quick to complete, as it involved assigning teeth to one of five tooth wear severity grades, all of which are textually defined and photographed. At the same time, potential for misinterpretation of these grades was repeatedly encountered during their assessment. This stems from some ambiguity in the definitions. For example, slight wear is defined as “polished surface and the dentine was exposed at the cusps”, moderate wear shows “dentine of teeth … showed greater and more even exposure”, and heavy wear is described as “teeth already irregularly worn” [56, p. 33]. These definitions made it difficult to readily differentiate between wear categories. Method C was more time-consuming to implement and also used vague language that made choosing the most appropriate age category challenging. For example, age five is defined as having “slightly more wear on the posterior half of the P_2_, the anterior and posterior buccal cusps of M_1_ and the distal cusp of M_3_” [61]. Terms such as “slightly more” leave a great deal of room for subjectivity. Additionally, descriptors such as “very worn” [p. 18], “horizontally inclined buccal-lingual plane” [p. 17], and “extensive attrition” [p. 18] are used but not defined, again likely resulting in more subjective assignments.

Greater accuracy is expected where more teeth are included in analysis, as more wear data is available per individual. However, excluding some teeth enhances efficiency in method application because there are fewer features to observe per individual. Each of the three methods incorporates post-canine teeth differently. Method A does not involve dP_2_, P_2_, dP_3_, or P_3_. Criteria for Method B rely more on molars than premolars, though premolars may be relevant in assigning age criteria at any age [56]. Method C does not include M_2_ in descriptions for ages five, six, and eight, and only generally describes wear for all molars together at ages three, four, and seven [61]. However, M_2_ wear severity was still graded for all individuals.

All methods take quite different approaches to characterizing wear. As mentioned above, Method A [60] is focused on two-dimensional visual representation of enamel and dentine shapes, an approach that has proven useful in wear studies with other taxa [5,12,15,86]. Method B [56] uses one series of relative tooth wear grades (none, slight, moderate, heavy, and very heavy) for all tooth types. Method C [61] relies heavily on text description, highlighting whether lines of dentine are narrower or wider than adjacent enamel, the mesial half of the P_2_ shows wear, particular cusps (especially on the M_3_) show wear, the pointed appearance of premolar cusps, the angle of the buccal-lingual plane or flattening of the occlusal surface, and extreme wear on the M_1_. These traits, while reasonably extensive, largely ignore the shape and connections of dentine and enamel that can be easily recognized. In contrast, occlusal surface angles play prominent roles in Method C, but these are difficult to observe in photos and likely undiscernible if only occlusal views are available. For younger individuals, all three methods focus on tooth eruption sequences which, in agreement with the lower error found in this study, provide a relatively low error for age estimation.

## Conclusion

Comparing known ages of caribou mandibles with estimated ages generated through three methods revealed the strengths and weaknesses of each approach. The two schemes of Method A, developed by van den Berg and colleagues [60], provide efficient and intuitive approaches. However, these two schemes do not allow for age to be estimated for the Canadian caribou populations represented in our sample. The Svalbard or Absolute scheme is intended only for Svalbard reindeer, a highly unique *Rangifer* population, and when used with this study’s sample (which is not recommended) underestimated the ages of most adults. The uncalibrated or Relative scheme used in Method A generates only relative age assessments. To be used to generate ‘absolute’ age estimates elsewhere will always require calibration using known-age specimens from relevant populations. This restrains its applicability to other *Rangifer* populations, virtually all of which have been used by people more extensively and for longer periods than those on the Svalbard Archipelago. At the same time, Method A is by far the most user-friendly approach tested in this study.

Method B, designed by Pasda [56], also provides an efficient process for visually grading tooth wear based on severity. However, this method results in age categories of varying lengths which overlap with one another, complicating interpretations of age-based data. This method also did not estimate any ages over seven years. Method C, by Miller [61], involved visually assessing mandibular tooth wear using text descriptions and reference photos to assign ages, which were then placed into recommended age categories. While these age categories were well-suited for tooth wear age estimation analysis, the procedure was less user-friendly than the other two methods and a relatively high number of individuals were incorrectly assigned. The strength of this study is its sample size, which dwarfs that of the other two studies.

Tooth wear age estimation is a valuable analytical tool for assessing the ages of *Rangifer* and other animals that were critical to past human populations. This study demonstrates a clear need for improved methodologies that are built from larger samples and that can be used on a greater breadth of *Rangifer* populations. Refined definitions of traits used in scoring wear also will be necessary, and ideally, both ‘absolute’ and relative age assessments will be possible. Such methodological developments would help strengthen archaeological interpretations of human involvement with *Rangifer* in the distant past, including as prey animals or domestic livestock. Just as importantly, a more widely applicable and reliable methodology of estimating age in *Rangifer* will also be of use in wildlife biology to characterize modern populations, which are under increasing threats.

## Supporting information

S1 Table Known and estimated ages of caribou mandibles. (XLSX)
